# Long-term effectiveness and cost-effectiveness of an 18-week supervised exercise program in patients treated with autologous stem cell transplantation: results from the EXIST study

**DOI:** 10.1007/s11764-019-00775-9

**Published:** 2019-07-08

**Authors:** Johanna M. van Dongen, Saskia Persoon, Gabrielle Jongeneel, Judith E. Bosmans, Marie José Kersten, Johannes Brug, Frans Nollet, Mai J. M. Chinapaw, Laurien M. Buffart

**Affiliations:** 1 Vrije Universiteit Amsterdam, Department of Health Sciences, Faculty of Science, Amsterdam Public Health Research Institute, Amsterdam, The Netherlands; 2 Amsterdam UMC, University of Amsterdam, Department of Rehabilitation, Amsterdam Movement Sciences, Amsterdam, The Netherlands; 3 Amsterdam UMC, Vrije Universiteit Amsterdam, Department of Epidemiology and Biostatistics, Amsterdam Public Health Research Institute, De Boelelaan 1089a, 1018HV Amsterdam, The Netherlands; 4 Amsterdam UMC, University of Amsterdam, Department of Haematology, Amsterdam, The Netherlands; 5 Lymphoma and Myeloma Center Amsterdam (LYMMCARE) and Cancer Center Amsterdam, Amsterdam, The Netherlands; 6 National Institute for Public Health and the Environment, Bilthoven, The Netherlands; 7 Amsterdam UMC, Vrije Universiteit Amsterdam, Department of Public and Occupational Health, Amsterdam Public Health Research Institute, Amsterdam, The Netherlands; 8 Amsterdam UMC, Vrije Universiteit Amsterdam, Department of Medical Oncology, Cancer Center Amsterdam, Amsterdam, The Netherlands

**Keywords:** Long-term effectiveness, Cost-effectiveness, Exercise intervention, Multiple myeloma, Lymphoma

## Abstract

**Purpose:**

To evaluate the long-term effectiveness and cost-effectiveness of a supervised 18-week high-intensity exercise program compared with usual care in patients treated with autologous stem cell transplantation.

**Methods:**

One hundred nine patients were randomly assigned to the exercise intervention (*n* = 54) or the usual care control group (*n* = 55). Data on cardiorespiratory fitness (VO_2_peak), handgrip strength, general fatigue, and health-related quality of life (quality-adjusted life years [QALYs]) were collected at baseline (T0), after completion of the exercise intervention or at a similar time point in the control group (T1) and 12 months later (T2). Cost questionnaires were used to assess societal costs. Long-term effectiveness (at T2) was evaluated using linear mixed model analyses. For the economic evaluation, missing data were imputed using multiple imputation, and data were analyzed using linear mixed models.

**Results:**

At T2, no statistically significant differences were found between the intervention and control group for VO_2_peak (0.12; 95%CI − 1.89; 2.14 ml/min/kg), handgrip strength (− 1.08; 95%CI− 2.47; 2.31), and general fatigue (− 0.69; 95%CI − 2.52; 1.14). During 12-months follow-up, no significant between-group differences in QALYs and societal costs were found (QALYs − 0.07; 95%CI − 0.17; 0.04; costs 529; 95%CI − 3205;4452). Intervention costs were €1340 per patient. For all outcomes, the probability of the intervention being cost-effective was low at reasonable values of willingness-to-pay.

**Conclusion:**

We found no evidence for the exercise intervention being effective on physical fitness and fatigue, nor cost-effective from a societal perspective.

**Trial registration:**

The study was prospectively registered on 27 May 2010 at the Netherlands Trial Register (NTR2341).

**Implications for Cancer Survivors:**

The current exercise intervention should not be recommended to patients recently treated with autologous stem cell transplantation.

## Introduction

Cancer and cancer treatment may have a negative impact on physical fitness, fatigue, and quality of life, which may be counteracted by exercise interventions. Several systematic reviews reported favorable effects of exercise interventions on cardiorespiratory fitness [4, 9, 28], muscle strength [9, 28], fatigue [4, 9], and health-related quality of life (HRQoL) [3, 30] in patients with cancer during and following cancer treatment. In various countries, this has resulted in the development of exercise guidelines for patients with cancer [2]. Beneficial effects on these outcomes were also found in patients who received a stem cell transplantation [20, 22].

A recent study has shown that the effects of exercise interventions following cancer treatment for solid tumors on physical fitness and HRQoL can be sustained at 1 year, while the intervention effects on fatigue disappeared [12]. To the best of our knowledge, effects of exercise interventions in patients who received a stem cell transplantation on these outcomes at long-term (≥ 1 year) are unknown.

Information on the cost-effectiveness of exercise interventions is important for decision-makers, as this provides insight into the additional cost per unit of effect gained, and may thus provide guidance to decision-makers when deciding how to allocate scarce resources in healthcare [7, 27]. Nonetheless, there is only limited evidence available on the cost-effectiveness of exercise interventions in patients with cancer. A previous systematic review on this topic [18] included only three studies. Since then, a small number of additional studies have evaluated the cost-effectiveness of exercise interventions during or after cancer treatment [1, 10, 12, 17, 19, 35], but they were heterogeneous in the type of cancer, type of intervention and control condition, study results, and/or they were not based on patient-level data. Given the importance of this topic and the scarcity of literature, more research on the cost-effectiveness of exercise interventions in patients with cancer is warranted.

Recently, we have published the results of the EXercise Intervention after Stem cell Transplantation (EXIST) randomized controlled trial [23]. Within this study, an 18-week supervised high-intensity combined resistance and interval exercise program was compared with usual care in patients with a hematologic malignancy treated with autologous stem cell transplantation. At the short-term (directly after the intervention), the exercise intervention did not have beneficial effects on physical fitness, fatigue, and HRQoL [23]. Some may argue that the exercise intervention will therefore not be effective, nor cost-effective at the long-term, and that publication of these results is therefore not necessary. However, the EXIST exercise intervention also included counseling sessions aiming to promote compliance and maintenance of exercise after the program [21, 23, 24] and it is currently unclear whether the intervention has delayed effects. Additionally, for cost-effectiveness analyses, it is important that the time frame of analyses extends beyond the intervention period to ensure that the most important costs and consequences flowing from the intervention are covered [7]. This is because patients with relatively large health effects directly after the intervention typically have relatively low levels of healthcare consumption and productivity losses during the consecutive period and vice versa. To cover this research gap, the present study aimed to evaluate the long-term effectiveness and cost-effectiveness of the exercise intervention versus usual care for physical fitness, fatigue, and HRQoL in patients treated with autologous stem cell transplantation.

## Methods

The EXIST study was a multicenter randomized controlled trial that evaluated the effectiveness of an 18-week supervised high-intensity combined resistance and interval exercise program compared with usual care on physical fitness and fatigue as primary outcomes as well as its cost-effectiveness in patients with a hematologic malignancy treated with autologous stem cell transplantation. The study procedures were approved by the Medical Ethics Committee of the Academic Medical Center (METC AMC 10/106) and by the boards of the Antoni van Leeuwenhoek Hospital (Amsterdam), St. Antonius Hospital (Nieuwegein), Haga Teaching Hospital (Den Haag), University Medical Center (Utrecht), Isala (Zwolle), Erasmus MC/Daniel den Hoed (Rotterdam), VU University Medical Center (Amsterdam), and Leiden University Medical Center (Leiden). The study protocol as well as the baseline and short-term results have been published previously [21, 23, 24].

Patients were recruited between March 2011 and February 2014. Patients were eligible for the study if they were (1) treated with autologous stem cell transplantation for multiple myeloma or lymphoma 6–14 weeks earlier or treated with autologous stem cell transplantation and finished successive consolidation chemotherapy or radiotherapy 2–6 week earlier; (2) recovered from treatment (Hb > 10.5 g/dL, platelets > 80 × 10^9^/L); and (3) able to undergo exercise testing and to participate in an exercise intervention. All patients provided written informed consent.

After the baseline assessments and confirmation of eligibility, patients were randomized to the intervention or usual care group by an independent data manager using a validated software program (TENALEA Clinical Trial Data Management System; Netherlands Cancer Institute, Amsterdam, the Netherlands). Randomization was concealed, stratified for transplant center and diagnosis using block randomization with block sizes varying randomly between 2, 4, and 6. The study personnel that assessed long-term outcomes and performed the analyses were not blinded for treatment allocation.

### Exercise intervention and usual care

The 18-week exercise intervention was supervised by instructed physical therapists and took place at local physical therapy practices. The program consisted of 30 exercise and 6 counseling sessions. Each exercise session lasted approximately 60 min and included 6 resistance exercises targeting the large muscle groups (vertical row, leg press, bench/chest press, and pull over/flies and two additional exercises for the abdominal muscles and the upper legs) and 2 bouts of 8 min cycling interval training (Table 1). The indirect one repetition maximum (1-RM) test [15] and the steep ramp test [5] were performed every 4 weeks in order to tailor and adjust the exercise intensity prescription. Counseling sessions lasted 5 to 15 min each and took place in week 1, 4, 10, 12, 18, and 22 [21]. The counseling sessions were provided by the physical therapist who supervised the exercise intervention and aimed to improve compliance to the exercise intervention and to encourage patients to pursue a physically active lifestyle during and following the program [21]. From week 12 onwards, patients were encouraged to meet physical activity levels as recommended by guidelines [11]. Specific program elements included the provision of general and motivational information, both verbally and via folders, about the desired frequency, duration, and intensity of physical activity [21].

**Table 1:** Structure of the exercise program

Week	Frequency of exercise session	Type of training	Frequency, intensity, and duration of the exercises/sessions
1–8	Twice a week	Resistance exercises	2 sets of 10 repetitions at 65–80% of 1-RM*
		Cycling interval training	2 × 8 min, alternating 30s at 65% and 60s at 30% MSEC
9–12	Twice a week	Resistance exercises	See week 1–8
		Cycling interval training	2 × 8 min, alternating 30s at 65% and 30s at 30% MSEC
12–18	Once a week	Resistance exercises	2 sets of 20 repetitions at 35–40% of the 1-RM*
		Cycling Interval training	See week 9–12
1, 4, 10, 12, and 18		Physical activity counseling	5–15 min per session

*1-RM* one repetition maximum, *MSEC* maximal short exercise capacity, i.e., the highest workload achieved during the steep ramp test

*For the two additional exercises the protocol included the performance of 2 sets of 0.7 times the maximal number of repetitions

Usual care varied according to patients’ and physicians’ preferences. Control group patients were not restricted in their physical activities or in their use of healthcare services.

### Timing of assessments

The effect measures were assessed at baseline (T0), after completion of the intervention or at a similar time point in the usual care control group (T1; ~ 22 weeks after T0), and 1 year after T1 (T2). Patients visited one of two test centers to participate in exercise testing and filled out questionnaires at home. Cost data were assessed using cost questionnaires: patients filled out five monthly cost questionnaires between T0 and T1, and four 3-monthly questionnaires in the year between T1 and T2. The physical therapists kept a training log during the intervention program.

### Effect measures

The primary effect measures were physical fitness, including cardiorespiratory fitness and handgrip strength, and general fatigue.

Cardiorespiratory fitness was expressed as the highest continuous 15 s interval values for oxygen uptake (VO_2peak_ in ml/kg/min**;** MasterScreen CPX, CareFusion, Hoechberg, Germany) measured during a cardiopulmonary exercise test performed on a cycle ergometer (Lode Excalibur, Groningen, the Netherlands).

Handgrip strength of the dominant hand was assessed using a grip strength dynamometer (Hydraulic Hand Dynamometer, North Coast Medical Inc., Morgan Hill, USA), and the highest score out of three attempts in kilogram was used in analyses.

General fatigue was determined using the subscale of Multidimensional Fatigue Inventory (MFI) questionnaire [29]. The patients could score between 4 and 20, with higher scores indicating more fatigue.

For the economic evaluation, HRQoL was assessed using the EQ-5D-3L [8]. The EQ-5D-3L was administered at T0, T1, T2, and in an additional cost diary half-way between T1 and T2. The EQ-5D-3L consists of five questions evaluating the following health dimensions: mobility, self-care, usual activities, pain/discomfort, and anxiety/depression. The patients’ EQ-5D-3L health states were transformed into utility scores using the Dutch tariff [14]. QALYs were calculated by multiplying the patients’ utility scores by the time they spent in a certain health state using linear interpolation between measurement points. More QALYs indicate a better HRQoL, with approximately 1.3 QALYs as the maximum number (i.e., 1.3 years in optimal health).

Effects on all primary outcomes and HRQoL occurring after 1-year follow-up were discounted at a rate of 1.5% [31].

### Measurement and valuation of resource use

Costs were measured from a societal perspective and included intervention costs, healthcare costs, costs of informal care, sports costs, unpaid productivity costs, and absenteeism costs. Clinical trial–related costs (e.g., costs related to the physical measurements) were not included.

Intervention costs were estimated using a micro-costing approach, meaning that detailed information was collected on the use of intervention-related resources as well as their unit prices. Information on all other kinds of resource use was collected using cost questionnaires administered on a 1-monthly basis between T0 and T1 and a 3-monthly basis between T1 and T2. To cover the complete duration of follow-up, cost questionnaires administered between T0 and T1 had a recall period of 1 month, while cost questionnaires administered between T1 and T2 had a recall period of 3 months. The cost questionnaire was developed by the research team in close collaboration with experts in health economic evaluations.

Intervention costs included all costs related to the implementation and execution of the exercise intervention, i.e., costs related to the training of the physical therapists, the provision of the exercise and counseling sessions, and the intervention materials. Time investments of the study team and physical therapists were valued using standard costs or, if unavailable, salary data derived from their respective Collective Labor Agreement [31]. Data on the attendance of exercise and counseling sessions were retrieved from the training logs and the associated correspondence between the study team and the physical therapists. Invoices were used to value material costs.

Healthcare costs included the costs of primary healthcare (i.e., general practice, physical therapist), secondary healthcare (i.e., outpatient care, hospitalization, professional home care), and prescribed as well as over-the-counter medication. Primary and secondary healthcare utilization were valued using Dutch standard costs [31]. If unavailable, prices of professional organizations were used. Medication use was valued using unit prices of the Royal Dutch Society of Pharmacy.

Informal care was defined as care provided by family and friends, and was valued using a recommended Dutch shadow price [31].

Sports costs included the patients’ expenses on sporting goods and sports memberships.

Unpaid productivity costs consisted of costs related to lost hours of domestic tasks, educational activities, and volunteer work. Unpaid productivity losses were valued using a recommended Dutch shadow price as well [31].

Absenteeism costs related to paid work were estimated using the friction cost approach (FCA). The FCA assumes that costs are limited to the period Dutch companies need to replace a sick worker (i.e. friction period), which was estimated to be 23 weeks at time the intervention was provided [31]. After truncating the number of sickness days at the friction period, sickness days were valued using age- and gender-specific price weights [31].

All costs were expressed in 2014 Euros using consumer price indices. Costs occurring after 1-year follow-up were discounted at a rate of 4% [31].

### Statistical analyses

Long-term effectiveness and cost-effectiveness analyses were performed in accordance with the intention-to-treat principle. Descriptive statistics were used to describe baseline characteristics of patients from the intervention and control group, and of patients with complete data and those with incomplete data.

Linear mixed model analyses were conducted to evaluate the intervention effects on cardiorespiratory fitness, handgrip strength, and fatigue at long-term (T2). The intervention was regressed on the outcome value at the short-term (T1) and long-term (T2) simultaneously, adjusted for baseline levels of the outcome variable in the model, age, gender, and education level. This procedure automatically takes into account missing values using maximum likelihood estimation. A random intercept for transplant center was added to take into account the clustering of patients within centers. Regression coefficients and 95% confidence intervals (CI) of clinical intervention effects at long-term were presented.

In the main cost-effectiveness analysis, patients with multiple myeloma using the extremely expensive drug lenalidomide (Revlimid) as maintenance treatment after autologous stem cell transplantation (i.e., about €100,000 per year) were treated as being lost to follow-up from the moment they started using lenalidomide. From that point onwards, all of their cost and effect measure values were set at missing and imputed using multiple imputation. This was done to prevent the cost estimates from being biased by the inclusion of these extremely high costs. This was considered to be an appropriate strategy, as we do not expect the exercise intervention to have an impact on a patient’s need for lenalidomide.

Missing data in the cost-effectiveness analysis were handled using multiple imputation by chained equations stratified by treatment group. The imputation model included variables that differed between patients with complete and incomplete data (i.e., education level, anti-cancer medication, and smoking), those predicting the “missingness” of data (i.e., age, gender, diagnosis, sport history, and time between the start of the study and autologous stem cell transplantation), baseline effect values and all available follow-up cost and effect values. Using predictive mean matching, twenty-five complete data sets were created in IBM SPSS resulting in a loss of efficiency smaller than 5% (v23.0, Chicago, IL). All twenty-five datasets were analyzed separately as specified below. Pooled estimates were subsequently calculated using Rubin’s rules [36].

For calculating ICERs, the mean difference in total costs between the intervention and control group was divided by the mean difference in effects, expressed in terms of cardiorespiratory fitness, grip strength, fatigue, and QALYs. Cost and effect differences were estimated using linear multilevel analyses, with a two-level structure: observations, transplant center. Within these analyses, cost and effect differences were corrected for age, gender, and education level. To deal with the highly skewed nature of cost data, joint uncertainty around costs and effects was estimated using the Bias Corrected Bootstrap method with 5000 replications. To graphically illustrate the uncertainty surrounding the ICERs, bootstrapped incremental cost-effect pairs were plotted on cost-effectiveness planes. A summary measure of the joint uncertainty of costs and effects was presented using cost-effectiveness acceptability curves (CEACs). CEACs indicate the probability of an intervention being cost-effective in comparison with the control condition for a range of willingness-to-pay values (i.e., the maximum amount of money decision-makers are willing to pay per unit of effect gained). Except for the multiple imputation, analyses were performed using Stata v12. Statistical significance was set at *p* < 0.05 [33].

To test the robustness of the results, four sensitivity analyses were performed. First, analyses were performed using the Human Capital Approach (HCA), instead of the FCA (SA1). The HCA regards each hour not worked by the patient as an hour of lost productivity. In contrast to the FCA, the HCA does therefore not truncate costs to the friction period. Second, analyses were performed in which intervention costs were calculated using a total number of training sessions per patient of 30 (SA2). This was done because the intervention was intended to last for 30 training sessions. In the third sensitivity analysis, only healthcare costs were analyzed (i.e., the healthcare/NHS perspective was applied)(SA3). In the fourth sensitivity analysis (SA4), no correction was made for the use of lenalidomide.

## Results

### Patients

**Fig. 1:**
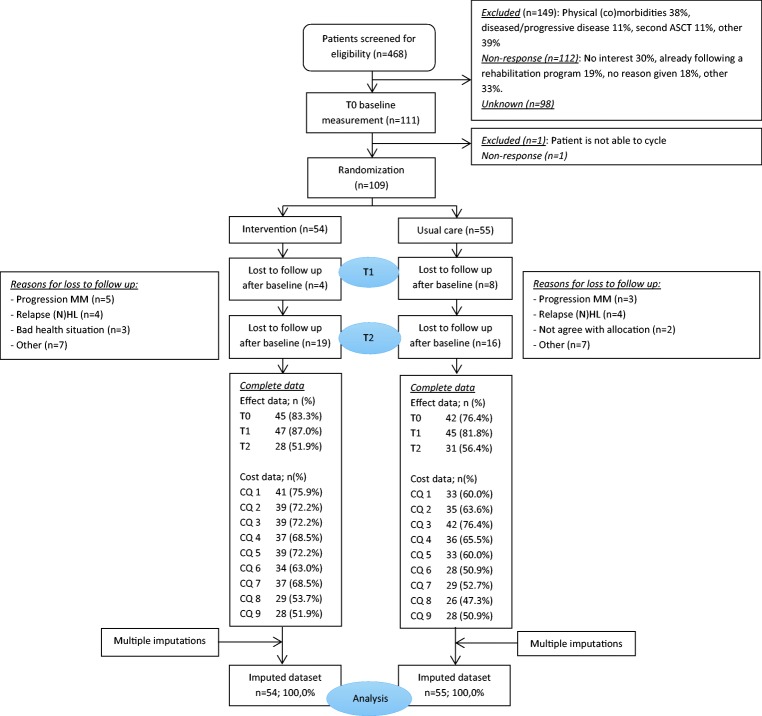
Flow diagram of patients in the EXIST study

In total, 109 patients were randomized to the intervention (*n* = 54) or control group (*n* = 55). At baseline, relevant differences were found between both groups for gender and education level (Table 2). The percentage of patients with complete effect and cost data at the different measurement points can be found in Fig. 1. Data on the total number of training sessions provided were complete. The main reasons for loss to follow-up were relapse of (non-)Hodgkin lymphoma or progression of multiple myeloma (Fig. 1). Differences in education level, smoking, and time since autologous stem cell transplantation were found between patients with complete and incomplete follow-up data (data not shown).

**Table 2:** Baseline demographics and clinical characteristics of the patients

	All (*n* = 109)	Intervention group (*n* = 54)	Control group (*n* = 55)
Male ([*n* (%))	69 (63)	32 (59)	37 (67)
Age (mean (SD))	52 (11)	52 (11)	53 (12)
Married/living together (*n* (%))	91 (84)	45 (83)	46 (84)
Higher education level (*n* (%))	39 (36)	15 (28)	24 (44)
Smoker (*n* (%))	14 (13)	7 (13)	7 (13)
Active^1^ (*n* (%))	69 (63)	33 (61)	36 (66)
Cancer type			
MM (*n* (%))	58 (53)	29 (54)	29 (53)
(N)HL (*n* (%))	51 (47)	25 (46)	26 (47)
Time since ASCT (mean (SD))	86 (45)	84 (46)	88 (43)
Number of co-morbidities (mean (SD))	2 (2)	2 (2)	2 (2)
VO_2_peak (mean (SD)) in ml/kg/min	22 (5)	21 (5)	22 (6)
Hand grip strength (mean (SD)) in kg	36 (11)	36 (12)	37 (10)
General Fatigue (mean (SD)) (range 0–20)	13 (4)	13 (4)	14 (4)

*n* number, *SD* standard deviation, *MM* multiple myeloma, *(N)HL* (non-)Hodgkin lymphoma, *ASCT* autologous stem cell transplantation

^1^Patients who participating in sports at least once a week before diagnoses/relapse

In total, 75% of the patients attended ≥ 80% of the training sessions and 87% of the patients attended the counseling sessions (Table 3) [25].

**Table 3:** Difference in pooled mean costs and effects (95% confidence intervals), incremental cost-effectiveness ratios, and the distribution of cost-effect pairs around the quadrants of the cost-effectiveness planes

	Sample size		Outcome	ΔC (95% CI) €	ΔE (95% CI) points	ICER €/point	Distribution CE-plane (%)			
	I	C								
							NE^1^	SE^2^	SW^3^	NW^4^
Main Analyses	54	55	Cardiorespiratory fitness	529 (− 3205 to 4452)	0.54 (− 1.14 to 2.23)	972	43.7	30.9	11.5	13.9
			Hand grip strength	529 (− 3205 to 4452)	− 1.24 (− 4.10 to 1.59)	− 427	10.8	8.9	33.5	46.8
			General Fatigue	529 (− 3205 to 4452)	− 1.78 (− 3.94 to − 0.37)	− 297	53.3	40.1	37.1	4.4
			HRQoL - QALY	529 (− 3205 to 4452)	− 0.07 (− 0.17 to 0.04)	− 8043	4.6	5.2	37.1	53.1
SA1 Human Capital Approach	54	55	Cardiorespiratory fitness	1152 (− 5585 to 7936)	0.54 (− 1.14 to 2.23)	2117	46.2	28.4	9.8	15.5
			Hand grip strength	1152 (− 5585 to 7936)	− 1.24 (− 4.10 to 1.59)	− 931	10.9	8.8	29.5	50.8
			General Fatigue	1152 (− 5585 to 7936)	− 1.78 (− 3.94 to − 0.37)	− 646	57.4	35.9	2.3	29.5
			HRQoL - QALY	1152 (− 5585 to 7936)	− 0.07 (− 0.17 to 0.04)	− 17,515	5.4	4.4	33.8	56.4
SA2 Maximal training sessions	54	55	Cardiorespiratory fitness	843 (− 2871 to 4779)	0.54 (− 1.14 to 2.23)	1549	48.3	26.4	9.9	15.4
			Hand grip strength	843 (− 2871 to 4779)	− 1.24 (− 4.10 to 1.59)	− 681	12.0	7.7	28.6	51.7
			General Fatigue	843 (− 2871 to 4779)	− 1.78 (− 3.94 to − 0.37)	− 473	59.0	34.4	1.9	4.7
			HRQoL - QALY	843 (− 2871 to 4779)	− 0.07 (− 0.17 to 0.04)	− 12,817	5.2	4.6	31.7	58.5
SA3 Medical costs only	54	55	Cardiorespiratory fitness	1096 (− 114 to 2220)	0.54 (− 1.14 to 2.23)	2016	70.7	4.0	0.8	24.6
			Hand grip strength	1096 (− 114 to 2220)	− 1.24 (− 4.10 to 1.59)	− 886	18.5	1.2	3.5	76.8
			General Fatigue	1096 (− 114 to 2220)	− 1.78 (− 3.94 to − 0.37)	− 615	88.8	4.6	1.3	6.5
			HRQoL - QALY	1096 (− 114 to 2220)	− 0.07 (− 0.17 to 0.04)	− 16,676	8.6	1.2	3.5	86.7
SA4 No correction for lenalidomide use	54	55	Cardiorespiratory fitness	15,646 (7688 to 30,845)	0.97 (− 0.74 to 2.69)	16,135	84.3	1.1	0.0	14.6
			Hand grip strength	15,646 (7688 to 30,845)	− 0.22 (− 2.96 to 2.51)	− 69,892	41.4	0.7	0.4	57.4
			General Fatigue	15,646 (7688 to 30,845)	− 0.94 (− 3.12 to 1.24)	− 12,667	80.0	1.0	0.1	18.9
			HRQoL - QALY	15,646 (7688 to 30,845)	− 0.07 (− 0.17 to 0.04)	− 242,963	10.6	0.1	1.0	88.2

*I* intervention group, *C* control group, *C* costs, *E* effects, *ICER* incremental cost-effectiveness ratio, *CE-plane* cost-effectiveness plane, *SA* sensitivity analyses, *QALY* quality-adjusted life years

Costs are expressed in 2014 Euros

1Refers to the northeast quadrant of the CE plane, suggesting that the EXIST exercise intervention is more effective and more costly than usual practice

2Refers to the southeast quadrant of the CE plane, suggesting that the EXIST exercise intervention is more effective and less costly than usual practice

3Refers to the northwest quadrant of the CE plane, suggesting that the EXIST exercise intervention is less effective and more costly than usual practice

4Refers to the southwest quadrant of the CE plane, suggesting that the EXIST exercise intervention is less effective and less costly than usual practice

### Long-term effectiveness: primary outcomes

At T2, no statistically significant differences were found between the intervention and control group for cardiorespiratory fitness (0.12 ml/kg/min; 95%CI − 1.89; 2.14, *p* = 0.90), handgrip strength (−0.08 kg; 95%CI − 2.47; 2.31, *p* = 0.95), and general fatigue (−0.69 points; 95%CI − 2.52; 1.14, *p* = 0.46).

#### Costs

On average, intervention costs were €1340 per patient (Table 4). During follow-up, secondary healthcare costs were statistically significantly higher in the intervention group than in the control group (Table 5). Unpaid lost productivity costs and informal care costs were statistically significantly lower in the intervention group than in the control group (Table 5). Total societal costs were higher in the intervention group than in the control group, but this difference was not statistically significant (€529; 95%CI − 3205 to 4452).

**Table 4:** Costs of the EXIST intervention per patient

	Staff	Units	Unit prices	Total costs	Total costs per patient
Kick-off physiotherapy (42 clinics)					
Information packet		42 packets	€1.97/packet	€82.59	€1.53
Information leaflet		42 leaflets	€0.83/leaflet	€34.78	€0.64
Time investment	Physical therapist	21 h	€48.81/h	€1025.02	€18.98
	Project assistant	21 h	€32.54/h	€683.35	€12.65
Traveling expenses		4451.2 km	€0.19/km	€858.63	€15.90
Physiotherapy					
Training sessions	Physical therapist	1356 h	€48.81/h	€66,171.60	€1225.40
Counseling sessions	Physical therapist	63.25 h	€48.81/h	€3087.27	€57.17
Consultation sports physician/intake	Sports physician	1 h	€91.91/h	€91.91	€1.70
	Physical therapist	1 h	€48.81/h	€48.81	€0.90
Information booklet		54 booklets	€3.90/leaflet	€210.60	€3.90
Registration logbook		54 logbooks	€1.16/logbook	€62.64	€1.13
Total					€1339.92

*km* kilometer

Costs are expressed in 2014 euros

**Table 5:** Mean costs per patient in the intervention and control group, and mean cost differences between both groups during follow-up

Cost category	Intervention group *n* = 54; mean (SEM)	Control group *n* = 55; mean (SEM)	Crude cost differences mean (95% CI)	Adjusted cost differences mean (95% CI)
Primary healthcare costs	1437 (225)	1955 (282)	− 518 (− 1280 to 154)	− 512 (− 1215 to 78)
Secondary healthcare costs	2338 (280)	1845 (198)	493 (−97 to 1235)	501 (97 to 1160)
Medication costs	1002 (135)	1271 (250)	− 269 (− 908 to 206)	− 237 (−884 to 246)
Unpaid productivity costs	560 (106)	1480 (276)	− 920 (− 1571 to 396)	− 884 (− 1437 to − 421)
Informal care costs	432 (122)	1148 (244)	− 716 (− 1298 to − 227)	− 669 (− 1159 to − 193)
Absenteeism costs	16,818 (1277)	15,823 (1294)	995 (− 2510 to 4550)	1160 (− 2114 to 4585)
Sport costs	470 (51)	596 (77)	− 126 (− 313 to 42)	− 111 (− 292 to 57)
Intervention costs	1340 (47)	0 (0)	1340 (1249 to 1432)	1344 (1243 to 1423)
Total costs	24,397 (1322)	24,119 (1430)	− 278 (− 4105 to 3548)	529 (− 3205 to 4452)

*n* number, *SEM* standard error of the mean, *CI* confidence interval, *SD* standard deviation

Costs are expressed in 2014 Euros

### Societal perspective: cost-effectiveness

**Fig. 2:**
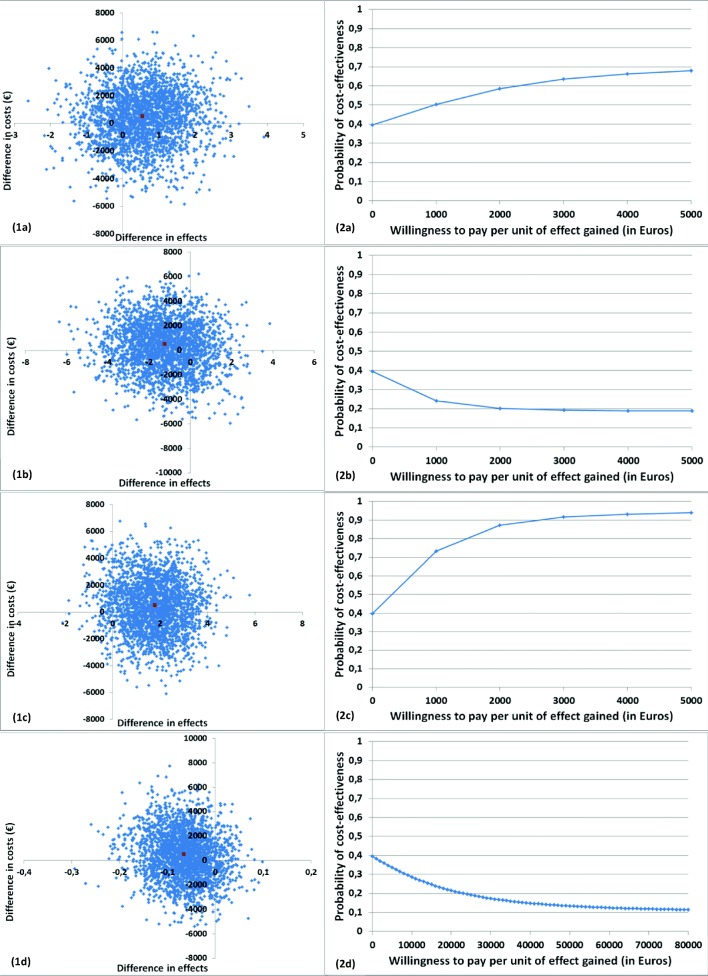
Cost-effectiveness planes indicating the uncertainty around the incremental cost-effectiveness ratios (1) and cost-effective acceptability curves indicating the probability of cost-effectiveness for different values (€) of willingness-to-pay per unit of effect gained (2) for cardiorespiratory fitness (a), handgrip strength (b), general fatigue (c), and health-related quality of life (d)

The ICER for cardiorespiratory fitness was 972, suggesting that an increase of 1 ml/kg/min in VO_2_peak in the intervention group was associated with a societal cost of €972 compared with the control group. The majority of cost-effect pairs were located in the northeast quadrant (i.e., 44%), indicating that the exercise intervention was on average more costly and more effective than usual care (Table 3; Fig. 2(1a)). The CEAC in Fig. 2(2a) indicates that the exercise intervention’s probability of being cost-effective compared with usual care was 0.39 at a willingness-to-pay of €0 per ml/kg/min, increasing to a maximum of 0.73 at a ceiling ratio of €20,000 per ml/kg/min.

The ICER for handgrip strength was − 427, indicating that the intervention was dominated by usual care (i.e., more costly and less effective)(Table 3; Fig. 2(1b)). The CEAC in Fig. 2(2b) indicates that the exercise intervention’s probability of being cost-effective compared with usual care was 0.39 at a willingness-to-pay of €0 per kg. At increasing levels of willingness-to-pay, this probability decreased.

The ICER for general fatigue was − 279. This suggests that a 1-point reduction in general fatigue in the intervention group was associated with a societal cost of €273 compared with the control group (Table 3; Fig. 2(1c)). Please note that a reduction in general fatigue indicates an improved health effect. The CEAC in Fig. 2(2c) indicates that the exercise intervention’s probability of being cost-effective compared with usual care was 0.39 at a willingness-to-pay of €0 per point improvement, increasing to a maximum of 0.96 at a willingness-to-pay of €31,000 per point improvement.

The ICER for HRQoL was − 8043, indicating that the intervention was dominated by usual care for QALYs (i.e., more costly and less effective)(Table 3; Fig. 2(1d)). The CEAC in Fig. 2(2d) indicates that if decision-makers are not willing to pay anything for a QALY gained, the probability of the intervention being cost-effectiveness was 0.39. At increasing levels of willingness-to-pay, this probability decreased.

### Sensitivity analyses

In SA1, SA2, and SA3, the total cost difference became larger compared with the main analysis, but remained in favor of the control group. In SA4, cost and effect difference estimates differed from those of the main analysis, with the most important difference being an almost 30-fold increase in the total societal cost difference (from €529 to €15,646).

## Discussion

Evaluations of the long-term effectiveness and cost-effectiveness of an 18-week supervised high-intensity combined resistance and interval exercise intervention versus usual care in patients after autologous stem cell transplantation presented in this article failed to show statistically significant benefits on physical fitness and general fatigue at long-term, and low probabilities of cost-effectiveness at reasonable values of willingness-to-pay. To illustrate the latter, at the lower and upper bound of the informal Dutch willingness-to-pay threshold (i.e., 10,000 and 80,000 €/QALY gained, respectively), the probability of the intervention being cost-effective compared with usual care was low (< 0.29).

The lack of significant between-group differences in physical fitness and fatigue at long-term is in line with previous studies in patients with breast cancer at 18 weeks [32] or 6 months follow-up [34]. However, a previous study among patients undergoing autologous or allogeneic stem cell transplantation including a 3-month follow-up showed benefits on cardiorespiratory fitness, assessed with a submaximal exercise test and knee extension strength, but not on handgrip and fatigue [13]. The lack of significant intervention effects at the long-term indicates that delayed effects of the intervention did not occur, and were comparable with the findings at short-term [23]. This may be related to the suboptimal timing of intervention delivery, as exercise post-stem cell transplantation may not be able to speed up natural recovery [23, 25]. A recent meta-analysis strengthens this hypothesis by reporting significant effects of exercise on fatigue and muscle strength pre-stem cell transplantation but not post-stem cell transplantation [16]. Other reasons for the lack of significance may be related to suboptimal exercise compliance in the intervention group or contamination in the control group [23], but no evidence for this hypothesis was found in a post hoc analysis [23] and process evaluation [25]. Finally, the lack of long-term effects also suggests that the counseling sessions alongside, and after, the supervised sessions were insufficient to change daily physical activity behavior. Due to practical reasons, physical therapists implemented the counseling sessions differently: some provided the counseling during the exercise sessions, while others made separate appointments [25]. Additionally, most were scheduled alongside the program to promote compliance and it might be that the three sessions focused on promoting physical activity in daily life were insufficient [21].

The finding that the supervised exercise intervention was not cost-effective versus usual care is probably due to the intervention not being effective at the short-term, nor at the long-term. The lack of cost-effectiveness is also in line with the results of an economic evaluation of a combined physical activity and dietary intervention in patients after autologous stem cell transplantation [10]. This study, however, used modeling techniques, rather than patient-level data. Patient-level studies evaluating the cost-effectiveness of exercise interventions for patients with solid tumors provided mixed results [1, 10, 12, 17, 19, 35]. Kampshoff et al. [12] found a 12-week high-intensity exercise intervention to be cost-effective compared with a moderate-intensity exercise intervention in a group of cancer survivors with solid tumors (mostly breast cancer), whereas May et al. [17] found an 18-week exercise program to be cost-effective versus usual care for colon cancer, but not for breast cancer. Van Waart et al. [34] found a low-intensity physical activity program not to be cost-effective compared with usual care among breast cancer patients undergoing chemotherapy, but found that a combined resistance and aerobic supervised exercise program may be considered cost-effective depending on the decision-makers’ willingness-to-pay.

Strengths of the study are the focus on the long-term (1 year after intervention completion) effectiveness and cost-effectiveness specifically in patients after autologous stem cell transplantation, the multicenter randomized controlled trial design, the use of a broad set of valid and reliable outcome measures, and the use of state-of-the-art statistical methods for evaluating the intervention’s cost-effectiveness (i.e., the use of seemingly unrelated regression, bootstrapping, and multiple imputation). Limitations of the study include the use of self-report for collecting cost data which may have introduced recall bias, the potential problems with generalizability to other countries with different healthcare systems and/or payment structures [6], and the large amount of missing cost data. However, missing effect and cost data were taken into account by the linear mixed model analyses and multiple imputation, respectively. Also, during follow-up, more intervention group patients used the extremely expensive drug lenalidomide than control group patients. As we did not expect the exercise intervention to have an impact on a patient’s need for lenalidomide, we dealt with this issue by treating patients as being lost to follow-up from the moment they started using lenalidomide, that is, as if their measurements were missing. The long-term effectiveness estimates slightly differ from those of the cost-effectiveness analyses. This is due to differences in the applied statistical methods, including (1) the use of mixed effect models in the effectiveness analyses, which are typically not applied in cost-effectiveness analyses, (2) multiple imputation, which is recommended for imputing missing cost data [26] versus maximum likelihood estimation, which is often used in effectiveness analyses, and (3) a correction for the possible correlation between costs and effects in the cost-effectiveness analyses [37].

The average cost of the 18-week exercise intervention of €1340 per patient on average was somewhat higher than the average cost of €858 per patient of the aforementioned 12-week high-intensity exercise intervention offered to patients with various types of cancer [12]. This amount is still relatively low as compared with the anti-cancer treatment costs. Therefore, exercise interventions, if effective, may have the potential to be included as part of standard care for patients with cancer. However, in its current form, and offered to patients recently treated with autologous stem cell transplantation, the exercise intervention is not effective at the short-term and long-term compared with usual care, nor cost-effective, and therefore, we do not recommend it to offer it as part of standard care. Future studies should examine the (long-term) effectiveness and cost-effectiveness of other types of exercise interventions and may concentrate on exercise interventions at different time points in the treatment trajectory of patients scheduled to receive stem cell transplantation.

In conclusion, we found no significant beneficial effects of the high-intensity combined resistance and interval exercise program on physical fitness and fatigue at long-term when compared with usual care, nor was the intervention cost-effective from a societal perspective.
